# Ability of Essential Oil Vapours to Reduce Numbers of Culturable Aerosolised Coronavirus, Bacteria and Fungi

**DOI:** 10.3390/antibiotics11030393

**Published:** 2022-03-15

**Authors:** Parthasarathi Kalaiselvan, Muhammad Yasir, Rajesh Kuppusamy, Mark Willcox, Ajay Kumar Vijay

**Affiliations:** School of Optometry and Vision Science, University of New South Wales, Sydney, NSW 2052, Australia; p.kalaiselvan@unsw.edu.au (P.K.); m.yasir@unsw.edu.au (M.Y.); r.kuppusamy@unsw.edu.au (R.K.); v.ajaykumar@unsw.edu.au (A.K.V.)

**Keywords:** aerosol, SARS-CoV-2 surrogate, essential oils, antimicrobial

## Abstract

Transmission of pathogens present in the indoor air can occur through aerosols. This study evaluated the efficacy of an evaporated mix of essential oils to reduce the numbers of culturable aerosolized coronavirus, bacterium and fungus. The essential oil-containing gel was allowed to vaporize inside a glass chamber for 10 or 20 min. Aerosols of a surrogate of SARS-CoV-2, murine hepatitis coronavirus MHV-1, *Escherichia coli* or *Aspergillus flavus* spores were produced using a collision nebuliser and passed through the essential oil vapours, then collected on a six-stage Andersen sampler. The six-stages of the impact sampler capture aerosols in sizes ranging from 7 to 0.65 µm. The number of culturable microbes present in the aerosols collected in the different stages were enumerated and compared to the number of culturable microbes in control microbial aerosols that were not exposed to the evaporated essential oils. After 10 and 20 min evaporation, the essential oils reduced the numbers of culturable aerosolized coronavirus by 48% (log_10_ reduction = 0.3; *p* = 0.002 vs. control) and 53% (log_10_ reduction = 0.3; *p* = 0.001 vs. control), respectively. The essential oils vaporised for 10 min, reduced the number of viable *E. coli* by 51% (log_10_ reduction = 0.3; *p* = 0.032 vs. control). The *Aspergillus flavus* spores were mostly observed in the larger aerosols (7.00 µm to 2.10 µm) and the essential oils vaporised for 10 min reduced the number of viable spores by 72% (log_10_ reduction = 0.6; *p* = 0.008 vs. control). The vapours produced by a gel containing naturally occurring essential oils were able to significantly reduce the viable numbers of aerosolized coronavirus, bacteria and fungal spores. The antimicrobial gel containing the essential oils may be able to reduce aerosol transmission of microbes when used in domestic and workplace settings.

## 1. Introduction

The majority of the urban population spend up to 90% of their time indoors [1,2]. The indoor environment harbours a diverse microbial population including viruses, bacteria, fungi and protozoa [3,4,5,6] that is referred to as the indoor microbiome. A major component of the indoor microbiome are endogenous microbes shed by human and animal occupants, with a minor constituent being the transient microbiota of the external environment transported inside [7]. Additional sources that can contribute to the indoor microbiome include water from indoor plumbing such as toilets and showers, soil, and heating and ventilation systems such as air-conditioning systems [4,8,9,10,11,12].

Human exposure to the indoor microbiome has been recognised as a factor for the development of respiratory diseases and allergies. Pathogens present in the indoor microbiome can be transmitted to humans either through aerosols or from contaminated surfaces. Key pathogens that are transmitted through aerosols include the bacteria *Staphylococcus aureus* [13], *Mycobacterium tuberculosis* [14], the fungus *Aspergillus fumigatus* [15], and the viruses influenza virus, Ebola and SARS-CoV [16]. While there was considerable speculation regarding the aerosol transmission of the SARS-CoV-2 virus [17,18], current data confirms aerosol transmission of this virus [19,20].

Hospitals and food industries use UV-C irradiation, plasma air ionization and fumigation with disinfectants to reduce air borne pathogens in the indoor air [21,22]. However, these strategies are expensive and may not be suitable in domestic settings. Portable indoor air cleaners/purifiers with HEPA filters are effective in reducing the microbial concentration in aerosols including SARS-CoV-2 in classrooms, offices and hospitals [23,24,25]. Vapours of essential oils have good antimicrobial activity against respiratory pathogens, including influenza virus [26,27] and offer an alternative strategy for disinfecting the indoor air [28,29,30]. The vapours, when dispersed in the air, can significantly reduce the microbial levels indoors [31,32,33]. A study has shown that vapours of the essential oils of cassia (*Cinnamomum cassia*) and clove (*Syzygium aromaticum*) can reduce the growth of *Salmonella enterica* serovar Typhi, *Yersinia enterocolitica* and *Escherichia coli* on agar plates [34]. Another study has demonstrated that vapours of the essential oils of red thyme can reduce the growth of fungi on agar plates [35].

Essential oils have been hypothesised to have anti-coronavirus activity, but this is based mostly upon molecular docking studies of the oils with surface spike proteins in SARS-CoV-2 [36]. They may also have effects on the envelop and capsid [37]. One study has found that several diterpenoids, sesquiterpenoids, triterpenoids and lignoids could reduce the cytopathic effect of SARS-CoV when coincubated with the cells and virus [38], probably by inhibiting a protease. Essential oils of *Laurus nobilis*, when added together with SARS-CoV, could reduce the infective titre of the virus in cell culture [39]. Essential oils from thyme can inhibit the replication of feline coronavirus when added shortly after infection of cells in laboratory studies [40]. Administration by spray of a mixture of oleoresins and essential oils from botanicals two hours prior to infection with the coronavirus avian infectious bronchitis virus decreased signs and symptoms and reduced the viral titre in chickens [41].

The current study aimed to evaluate the antimicrobial efficacy of vapours of an antimicrobial gel containing essential oils for its activity against aerosolised cells of a coronavirus surrogate of SARS-CoV-2, pathogenic bacteria and fungal spores.

## 2. Results

### 2.1. Analysis of the Essential Oils within the Gel Vapors

The total volatiles were 53.5 ± 0.3%. As the antimicrobial gel containing the essential oils was a commercial product, the identity of the essential oils it contains was unknown. However, experiments were performed using NMR and GCMS to preliminarily identify the essential oil compounds that were released. The essential oils that vaporized from the gel were preliminarily identified as eucalyptol, myrcene, limonene, terpinene and cymene.

### 2.2. Activity of the Gel in Solution against Coronavirus

The antimicrobial gel, containing the essential oils as active ingredients, when incubated in media with the coronavirus, reduced the numbers of infectious MHV-1 in a dose-dependent manner. The greatest quantity (50 mg) of the gel reduced the ability of the coronavirus to infect the mouse A9 cells by >99.99% (no viral cells were cultured) within 30 min of incubation compared to control (*p* < 0.001; Table 1). The smaller quantity (25 mg) of the gel reduced the numbers of coronavirus by 98.6% after 30 min of incubation compared to control (*p* < 0.001). There was no effect of incubation time for the 25 mg or 50 mg of the gel on virus numbers (*p* > 0.05).

### 2.3. Activity of the Evaporated Essential Oils against Coronavirus Aerosols

The essential oil vapours were active against MHV-1 aerosols. Initial studies using the vaporized essential oils without MHV-1 showed that the collected vapours that had dissolved into the DMEM or DMEM containing 20% BSA had no cytotoxic effect on the A9 cells.

**Figure 1:**
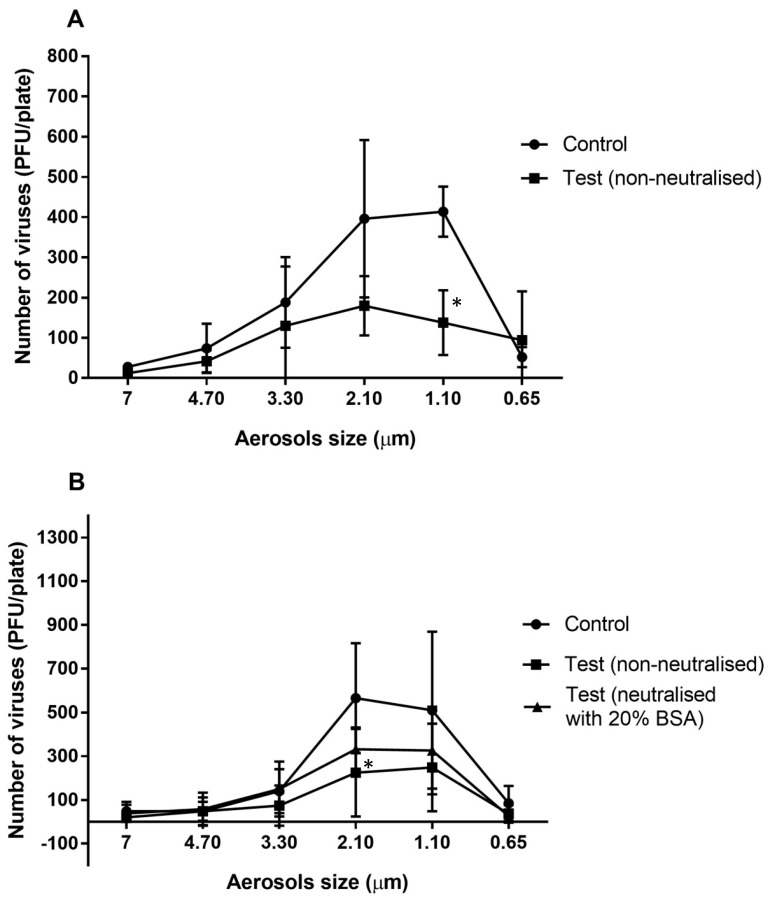
Number of murine hepatitis viral cells (MHV-1) recovered from different aerosol sizes with or without neutralisation of the vaporised essential oils for 10 min (**A**) or 20 min (**B**). The antimicrobial gel significantly (*) reduced the ability of viral aerosols to infected A9 cells in aerosols sizes of 2.10 and 1.10 µm compared to the untreated control (*p* = 0.011). Data points represent the mean (±95% confidence interval) of three independent experiments. BSA = bovine serum albumin.

The majority of the viral particles travelled in aerosols of 3.30 to 0.65 µm in the absence of the essential oil vapours (Figure 1A) with most viral particles travelling in the 2.10 and 1.10 µm aerosols (Figure 1A). After allowing the essential oils to vaporize in the chamber for 10 min, the numbers of viral particles that were able to infect the mouse cells were reduced for most aerosol sizes, with a significant reduction of 67% in the 1.10 µm aerosol (*p* = 0.011; Figure 1A). A slightly greater reduction of 78% was produced in the 2.10 µm aerosols compared to the controls when the essential oils were allowed to vaporize for 20 min (*p* = 0.011; Figure 1B). Overall, exposure of MHV-1 aerosols to the essential oil vapours (vaporized for 10 min) resulted a significant 48% reduction compared to the untreated control (*p* = 0.002; Table 2).

After allowing the essential oils to vaporize for 20 min, there was a significant 53% reduction in the number of viable aerosolized viral particles compared to the control (*p* = 0.001; Table 2). Following use of 20% BSA, the activity of the vapours of the essential oils was slightly but not significantly (*p* = 0.078; Table 2) reduced, resulting in a 33% reduction in the viability of viral aerosols compared to control (*p* = 0.001; Table 2)

### 2.4. Neutralization of Essential Oils

**Figure 2:**
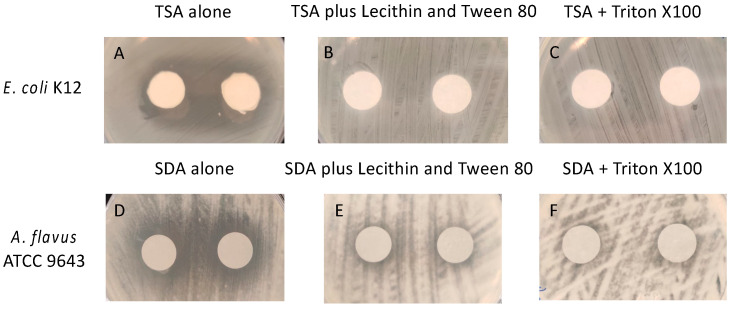
Demonstration of neutralization of active ingredients (essential oils) in the gel. (**A**,**D**) in the absencde of neutralisers, (**B**–**F**) in the presence of neutralisers. TSA = tryptic soy agar; SDA = Sabouraud’s dextrose agar.

In order to determine whether the essential oils (active ingredients) in the gels were biocidal or biostatic, experiments were conducted to find suitable neutralising agents. Figure 2 shows pictures of the zones of inhibition produced by the essential oils in the gel. Zones of inhibition of the growth of *E. coli* were produced by the essential oils (Figure 2A) and these were completely absent in the presence of Tween 80 + lecithin (Figure 2B) or Trition X100 (Figure 2C). Zones of inhibition were also seen with *A. flavus* (Figure 2D), and these were substantially reduced by both Tween 80 + lecithin and Trition X100 (Figure 2E,F).

### 2.5. Activity against Aerosols of Bacteria or Fungal Spores

**Figure 3:**
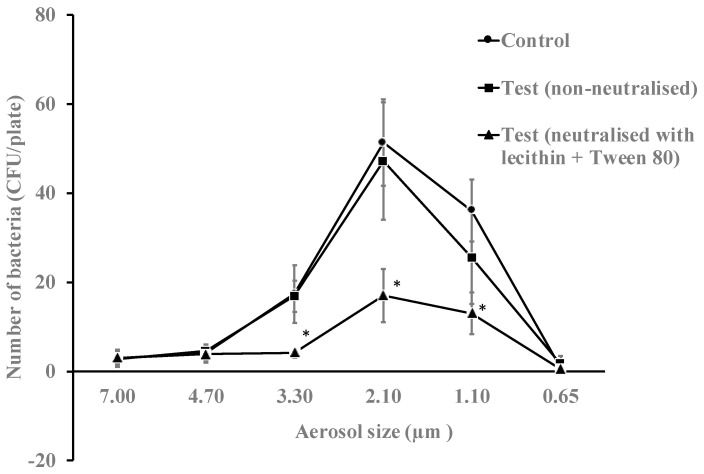
Numbers of *E. coli* K12 recovered from different aerosol sizes with or without neutralisation of the vapours of essential oils for 10 min. The gel significantly reduced the viability of bacteria in aerosols sizes 3.30, 2.10 and 1.10 µm when essential oils were non-neutralised during bacterial growth compared to the untreated control (*, *p* = 0.001). Data points represent the mean (±95% confidence interval) of three independent experiments. CFU = colony forming units.

The antimicrobial essential oils from the gel in vaporized form were active against aerosols of *E. coli*. In the absence of antimicrobial gel, this bacterium mostly travelled in aerosol particle sizes between 3.30 to 1.10 µm (Figure 3). Overall, the vapours of essential oils produced a reduction in bacterial viability of 29% (*p* = 0.018) when neutralised during bacterial growth and 51% (*p* = 0.032) when not neutralised during bacterial growth (Table 3). Without neutralising the gel during bacterial growth, the vapours of essential oils reduced the number of live bacteria in the 3.30, 2.10 and 1.10 µm aerosols by 76%, 69% and 64%, respectively (*p* = 0.001).

**Figure 4:**
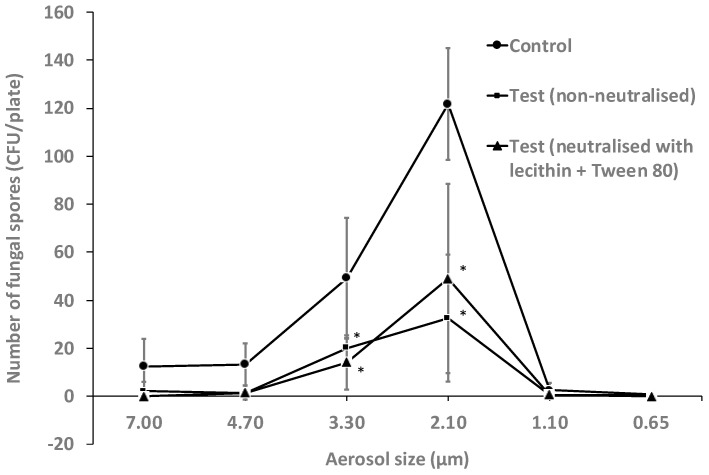
Number of *A. flavus* spores recovered from different aerosol sizes with or without neutralisation of the vaporized essential oils for 10 min. The essential oils significantly reduced the viability of spores of *A. flavus* in aerosols sizes 3.30 and 2.10 µm in both neutralised and non-neutralised conditions compared to the untreated control (*, *p* < 0.05). Data points represent the mean (±95% confidence interval) of three independent experiments. CFU = colony forming units.

Similarly, the essential oil vapours were also active against aerosols of *Aspergillus flavus* spores. The spores mainly travelled in aerosols of between 7.00 µm to 2.10 µm, with significantly (*p* < 0.001) higher numbers in 2.10 µm than other aerosols sizes (Figure 4). No spores travelled in aerosols of 1.10 µm or 0.65 µm (Figure 4). Overall, the vapours of essential oils reduced the viability of spores of *A. flavus* by 72% in non-neutralised conditions and 67% when neutralised (*p* ≤ 0.008; Table 4). Following exposure to the vapours of essential oils, the number of spores in aerosols of 2.10 µm was reduced compared to control by 60% and 73% in neutralised and non-neutralised conditions, respectively (*p* = 0.001). There was no effect of the addition of neutralising agents on the activity of the vapours of essential oils against *A. flavus* spores (*p* ≥ 0.08).

## 3. Discussion

This study has demonstrated that compounds found in essential oils composed of a mixture of eucalyptol, myrcene, limonene, terpinene and cymene have good antimicrobial activity against aerosols of coronavirus, bacteria and fungal spores. Direct contact with the essential oils in the gel resulted in a complete kill of the coronavirus. The essential oil vapours were able to reduce the numbers of aerosolised the coronavirus MHV-1 and the bacterium *E. coli* by ≥50% and the reduce the number of aerosolised *A. flavus* spores that could germinate by ≥66%.

SARS-CoV-2 is mainly spread via aerosols [20], therefore the ability of substances to prevent subsequent growth of the virus from aerosols might be important to reduce the spread of this disease. A previous study has demonstrated that aerosolized tea tree oil (from *Melaleuca* sp.) or eucalyptus oil can inactivate aerosolized influenza virus in a concentration and time dependent manner, with viral titres being uncountable within 30 min of exposure [27,42]. An aerosol of an essential oil blend of tea tree (*Melaleuca* sp.), eucalyptus and lemon myrtle in the ratio 4.5:4.5:1 has been shown to reduce the number of viable *E. coli* in aerosols to < 10% of the initial inoculum in 15 min, the number of aerosolized bacteriophage MS2 to < 10% after 60 min, and the number of *Aspergillus niger* spores to <10% in 120 min [43]. The analysis of compounds in the gel analysed in the current study revealed that it may contain terpene compounds such as limonene (from citrus), myrcene (from cannabis), cymene (from cumin and thyme) and terpinene (from cardamom and marjoram), as well as terpenoids such as eucalyptol (from eucalyptus). All these terpenes and terpenoids have been shown to posses antibacterial activity [44], and there is evidence that lower doses are needed to disrupt bacterial cell membranes (i.e., a synergistic activity) when used in combination [45].

Similarly to the current study when the essential oils in the gel were directly mixed with viruses in suspension, a nasal spray containing anise oil, eucalyptus oil, levomenthol, myrrh extract, clove oil, peppermint oil, ratanhia root extract and tormentil root extract, reduced the infectivity of SARS-CoV-2 when mixed in suspension [46]. 3β-Friedelanol, when mixed in media and applied to mammalian cells before viral infection, can prevent infection of cells by human CoV-229E [47]. A proprietary mix of essential oils has been shown to reduce the infectivity of surface dried bacteriophage phi 6 [48]. Another study reported that aerosolised influenza virus or bacteriophage M13 exposed to vaporised essential oils of tea tree or eucalyptus for 24 h resulted in approximately 87% reduction in influenza viral titres, but only 25–42% reduction in M13 titres [27]. The use of bacteriophages has been claimed to be a surrogate of coronaviruses; however, bacteriophages are structurally and biochemically very different to enveloped RNA viruses such as coronaviruses as they do not normally possess a lipid envelop that surrounds their capsid. Surface coatings composed of tea–cinnamaldehyde–copper or tea–cinnamaldehyde–silver can prevent infection of cells by the coronavirus MHV-A59 [49].

The essential oil vapours were bacteriostatic as their activity was diminished in the presence of agents that neutralized their antimicrobial compounds, while the viral and fungal activity was unaffected by neutralizers (20% BSA or lecithin/Tween 80, respectively). In vitro studies performed using essential oils have shown that the active compounds in essential oils are bactericidal for *E. coli* at higher concentrations and bacteriostatic at lower concentrations [50]. Essential oil vapours can affect spore formation in *A. fumigatus* [51] and can be either fungistatic or fungicidal depending on the active compound [52]. There are several potential chemicals that are recommended by ASTM International as appropriate to test for their ability to neutralize antimicrobial agents (see ASTM E1054-08, 2013 edition), and the list includes lecithin and Tween 80 that can be used to neutralize cresols and parabens that are chemically similar to essential oils present in the ingredients of used in the present study.

This study used a ready-made bacterial filtration efficiency testing rig that is usually used to assess the ability of face masks to filter the bacterium *Staphylococcus aureus* as specified by standard ASTM F2101-1 [53]. The Andersen impactor has been widely used to sample environmental bacteria and fungi [54] and has the advantage of being able to directly capture the biological aerosols on agar plates which can then be incubated to directly culture the organisms. Neutralizing chemicals can also be incorporated into the agar to inactivate antimicrobial agents present in the aerosols. While viruses can be captured on the agar plates, they had to be recovered from the agar and cultured on susceptible cells. Other researchers have used this method to culture virus in aerosols [55,56]. A major advantage of the Andersen impactor is that it allows differentiation of the size of the aerosols in which microbes travel [57].

Human activities such as speaking, coughing and sneezing generate microbial aerosols in sizes ranging from <1 µm to >100 µm [58,59,60,61]. Larger aerosols or droplets remain airborne for a short time and settle close to the source [62]. Smaller aerosols under 5 µm in size can remain airborne for longer periods and are able to make their way to the lungs [63]. Aerosols of this size are implicated in the airborne transmission of *Mycobacterium tuberculosis* [64], *Aspergillus fumigatus* spores [65], and viruses including the influenza virus [66] and SARS-CoV [67]. The present study demonstrated that vapours of essential oils could be active on aerosols of 7–1.1 µm.

The number of viral copies that are needed to cause an infection is expressed as ID_50_ which denotes the mean dose that causes an infection in 50% of susceptible subjects. While the ID_50_ for SARS-CoV-2 is not known, the ID_50_ for SARS-CoV ranged from 16 to 160 viral particles/person [68]. The essential oil vapours produced by the antimicrobial gel were able to significantly reduce the number of viable viral particles in aerosols under 5 µm by 48% within 10 min and the reduction increased to 53% when the gel was allowed to vaporise for 20 min indicating sustained and perhaps increasing antimicrobial activity. Allowing the gel to vaporise for longer durations and reducing the air flow (i.e., increasing the time for the virus and vapour to interact) may result in further reductions in viral numbers and this should be tested in future experiments.

We believe this report is the first to show activity of essential oil vapours against an aerosolized coronavirus. This study also demonstrated that essential oil vapours can significantly reduce the viable numbers of bacteria and fungal spores. Activity was rapid, occurring within the 2 min of air collection through the tube for viruses and fungal spores as the active ingredients in the essential oil vapours were neutralised during collection. Future work should examine the spectrum of activity of essential oil vapours in general, as well as those contained in the product used in the current study. Bioactive compounds in the essential oils at higher concentrations compared to the gel used in this study may impact human health [69]. Whilst the low concentration of the active compounds present in the gel in the current study may not impact human health, this should be examined in future studies. The results of the current study suggest that using essential oil vapours may reduce the transmission of respiratory pathogens, improve indoor air quality and the health of human occupants.

## 4. Materials and Methods

### 4.1. Microorganisms and Their Preparation

The mouse hepatitis virus (MHV-1) ATCC/VR261 is an enveloped single-strand RNA virus and an accepted surrogate of the SAR-CoV-2 virus (https://www.tga.gov.au/surrogate-viruses-use-disinfectant-efficacy-tests-justify-claims-against-covid-19; accessed on 7 January 2022) [70]. Viral stock was prepared by growing in A9 mouse fibroblast cells (ATCC/CCL 1.4) in Dulbecco’s minimum essential medium (DMEM, Thermofisher, Macquarie Park, NSW, Australia) containing 10% foetal bovine serum (FBS; Thermofisher), 100 µg/mL streptomycin sulphate and 100 I.U. penicillin G, (Thermofisher). Viral titres (1.0 × 10^5^ to 1.0 × 10^6^ plaque forming units (PFU)/mL) were determined by plaque assay. Aliquots were diluted ten-fold and inoculated into the wells of 12-well plates containing A9 cells and incubated for 1 h at 37 °C in the presence of 5% (*v*/*v*) CO_2_. The plates were gently rocked once every 15 min to prevent the cells from drying out. After incubation, an overlay media containing a 50:50 mix of 2% (*w*/*v*) agar (Sigma-Aldrich, Castle Hill, NSW, Australia) and DMEM was added to each well and further incubated for 72 h. Following incubation, the cells were fixed with 4% (*v*/*v*) formaldehyde (Sigma-Aldrich) for 2–3 h, the agar overlay removed, and the number of plaques produced by viral particles (PFUs) visualized after staining with 1% (*w*/*v*) crystal violet (Sigma-Aldrich).

*Escherichia coli* K12 (ATCC 10798) was grown overnight in tryptic soy broth (TSB; BD, Sydney, NSW, Australia) to mid-log phase. Following incubation, bacterial cells were collected by centrifuging and were washed once with phosphate buffer saline (PBS; NaCl 8 g L^−1^, KCl 0.2 g L^−1^, Na_2_HPO_4_ 1.15 g L^−1^, KH_2_PO_4_ 0.2 g L^−1^, pH 7.4). Following washing, cells were re-suspended in PBS and the concentration adjusted spectrophotometrically to an optical density of 0.1 at 660 nm which yielded 1.0 × 10^8^ colony forming units (CFU/mL) upon retrospective agar plate counts, then further serially diluted to a final concentration of 1.0 × 10^4^ CFU/mL. *E. coli* has been previously used in aerosol research as it can be aerosolised during use of toilet facilities [71].

The spores of *Aspergillus flavus* ATCC 9643 were produced by growth on Sabouraud’s dextrose agar (SDA; Thermofisher) for 10 days at 25 °C. The fungal growth was suspended in sterile deionized water and filtered through sterile 70 µm filters to remove hyphal fragments. Spores were resuspended in sterile deionized water and their concentration adjusted spectrophotometrically to an optical density of 0.2 at 660 nm which yielded 1.0 × 10^6^ CFU/mL, which were then serially diluted to a final concentration of 1.0 × 10^4^ CFU/mL.

### 4.2. Essential Oil Formulation

The essential oil-containing gel (Mould Gone, SAN-AIR, West Gosford, NSW, Australia) was supplied in sealed containers. The total volatile content of the sample was determined in duplicate in accordance with ASTM D 2369 “Volatile Content of Coatings” by heating the gel in a constant temperature oven held at 110 ± 5 °C. To identify the volatiles, the gel was kept in a closed glass jar and allowed to vaporize for 40 min. To enhance the release of the volatile compounds, the jar was kept in water bath with 50 °C. The vapour that settled on the side of jar was collected in deuterated chloroform (CDCl_3_) for 1H NMR analysis and compared with a control of CDCl_3_ alone. The types of volatile organic compounds present in the gel were examined by GC-MS with a modification of ASTM D6886-12 “Method for Low VOC Waterborne Coatings” using an injection temperature of 30 °C, an initial oven temperature of 30 °C that was increased to 80 °C over 10 min, sampling time 1.00 min, pressure 100.0 kPa (hold time 20 min), carrier gas hydrogen, total flow of 50.0 mL/min, column flow at 1.88 mL/min, linear velocity of 49.1 cm/s, and purge flow of 3.0 mL/min. The mass observed in the GC-MS was corelated with the 1H NMR data to give presumptive identification of the essential oils.

### 4.3. Activity of the Essential Oil-Containing Gel against Coronavirus in Solution

In order to demonstrate that the gel had anti-coronaviral activity, the first experiments incubated aliquots of the gel directly with viral particles in suspension. Cells of MHV-1 (1.0 × 10^5^ (PFU)/mL) were incubated with 25 mg or 50 mg of the antimicrobial gel in DMEM at ambient temperature for 0.5 or 2 h. Following incubation, the DMEM was removed, diluted in 20% (*w*/*v*) bovine serum albumin (BSA; Sigma-Aldrich) prepared in PBS and incubated for 10–15 min to neutralize the antimicrobial agents released from the gel. Thereafter, 100 µL aliquots were diluted ten-fold (in 20% BSA) and numbers assayed using the plaque assay as described above. Controls were the viral inoculum incubated in DMEM or PBS without the antimicrobial gel. The percentage reduction in PFU for each quantity of the gel compared to the negative control (PBS) was calculated.

### 4.4. Activity of the Essential Oils as Vapours against Coronavirus Aerosols

**Figure 5:**
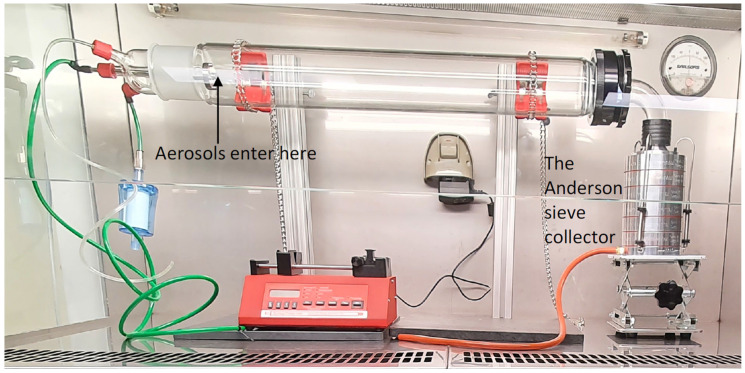
The bacterial filtration efficiency rig containing an Anderson sieve sampler. Aerosols of 3.0 ± 0.3 µm on average of viruses, bacteria or fungal spores were produced in the chamber.

A bacterial filtration efficiency (BFE) test rig (CH Technologies, Westwood, NJ, USA) was used to produce viral aerosols (Figure 5). The antimicrobial gel (10 g in total) was removed from its container and allowed to vaporise into the glass aerosol chamber (60 × 8 cm; 3016 cm^3^) for 10 min or 20 min prior to the introduction of the virus. The viral inoculum (50 µL; 1.0 × 10^6^ PFU/mL) was aerosolized using a continuous drive syringe pump through a nebulizer with an airflow of 28.3 L min^−1^ for one minute and allowed to interact with vapours of the antimicrobial gel as they passed through the glass tube. The size of the aerosols produced was approximately 3.0 ± 0.3 µm and these travelled through the glass aerosol chamber into an Anderson sieve sampler and were collected by flowing past 2% (*w*/*v*) agar plates. The largest (7 µm) sized aerosols were captured on the agar plate at the top of the Anderson sieve and the smallest (0.65 µm) on the agar plate at the bottom of the device. After one minute, the airflow was stopped to cease aerosol generation, and the vacuum pump was run for a further one minute to collect any residual aerosols from the glass chamber.

Following this, agar plates were flooded with 1.5 mL of either 20% BSA in DMEM (neutralised samples) or DMEM alone (non-neutralised samples), and viruses were carefully removed using a sterile cell scrapper. Aliquots (100 µL) from each plate were placed in duplicate on A9 cells in 12-well cell culture plates to culture any virus particles. The culture conditions were as described above. Control runs were performed at the beginning of each experiment prior to the addition of the gel in the glass aerosol chamber to collect infectious viruses so that any reduction in the number of infectious viruses could be calculated as a percentage of this control. In addition, controls to examine any cytotoxic effect of the essential oil vapours on the A9 cells were also examined.

This followed the protocol for viral testing. Briefly, the essential oils were allowed to evaporate from the gel for 20 min, after which time sterile DMEM was aerosolised into the chamber with an airflow of 28.3 L min^−1^ for one minute. After one minute, the airflow was stopped to cease aerosol generation, and the vacuum pump was run for a further one minute to collect any residual aerosols from the glass chamber. The aerosols were collected into the Anderson sieve containing agar plates. The plates were flooded with 1.5 mL of DMEM alone, scrapped to mimic the technique for viral collection, and then 100 μL samples added to A9 cells in 12-well culture plates. After incubation for 1 h at 37 °C in the presence of 5% (*v*/*v*) CO_2_ with rocking, overlay media (50:50 mix of 2% (*w*/*v*) agar and DMEM) was added to each well and further incubated for 72 h. Following incubation, the cells were fixed with 4% (*v*/*v*) formaldehyde for 2–3 h, the agar overlay removed, the cells stained with 1% (*w*/*v*) crystal violet and any cytotoxic effect examined by microscopy.

### 4.5. Activity Essential Oil Vapours against Bacterial and Fungal Spore Aerosols

Initially, the ability of two different potential neutralising agents were examined to determine which would neutralise the antibacterial and antifungal effects of the essential oil vapours. Tryptic soy agar (TSA; BD, Macquarie Park, NSW, Australia) or SDA was made containing either Tween^®^ 80 (5 mL L^−1^) and lecithin (0.7 g L^−1^), or 2% (*w*/*v*) Triton X100 (Sigma Aldrich, Castle Hill NSW, Australia). Filter paper discs (3 cm in diameter) were soaked whilst pre-heating (80 °C; 10 g liquified) the San Air gel. This allowed the assay to be performed in the absence of the gel itself, which may have had some activity which would not be present in the gel vapours. Lawns of *E. coli* (1 × 10^8^ mL^−1^) or *A. flavus* (1 × 10^6^ mL^−1^) were made of TSA or SDA plates, respectively, and then two paper discs soaked in San Air gel applied per plate. The plates were incubated for 16 h at 37 °C for the bacteria and 48 h at 25 °C for the fungi. After incubation, the size of zones of inhibition were compared.

The anti-bacterial activity of the essential oil vapours for 10 min against *E. coli* and its sporicidal activity against *A. flavus* spores was determined using a similar method as described for MHV-1, except using 50 µL of *E. coli* or *A. flavus* spores (1 × 10^4^ CFU/mL). Bacteria were collected on agar plates composed of tryptic soy agar (TSA; BD, Macquarie Park, NSW, Australia) alone or containing TSA and the neutralizers Tween^®^ 80 (5 mL L^−1^) and lecithin (0.7 g L^−1^). Fungal spores were collected on SDA plates alone or containing the same neutralizers. The numbers of viable cells from each of the six plates in the Anderson sieve collector were enumerated following incubation at 37 °C for 24 h for bacteria and at 25 °C for 72 h for fungal spores. Control runs were conducted prior to the addition of the gel in the glass aerosol chamber to collect viable bacteria and fungal spores. Test and control runs were performed in duplicate and repeated twice. The percentage of cells remaining viable after passage through the gel vapours was calculated by comparing numbers in the absence (control) and presence (test) of the gel vapours.

### 4.6. Statistical Analysis

Statistical analyses were performed using GraphPad Prism 7.04 software (GraphPad Software, La Jolla, CA, USA). The concentration and time dependent effect of the antimicrobial gel in solution was determined using two-way ANOVA. The effect of antimicrobial gel vapours at single time points on different aerosols sizes and overall percentage (%) reduction was assessed using Welch’s t-test and one-way ANOVA with Tukey’s test, respectively. Statistical significance was set as *p* < 0.05.

## 5. Conclusions

This study has demonstrated that short interactions between aerosolized coronavirus, the bacterium *E. coli* or spores of the fungus *A. flavus* and essential oil vapours from *Melaleuca* genus plants can reduce the number of culturable microbial cells. This may have implications in the control of airborne diseases such as COVID-19. The testing set up can be used in future studies to determine the ability of other essential oil vapours to reduce the viability of other microbes that can be transmitted via aerosols, such as *Mycobacterium tuberculosis*, *Legionella pneumophilia* and influenza viruses.

## Figures and Tables

**Table 1:** Effect of different concentrations of essential oil-containing gel against coronavirus in solution at two time points.

Sample	Amount of Gel (mg)	Incubation Time (min)	Number of Plaque Forming Units (PFU/mL)	Log_10_ Reduction	% Reduction	*p*-Value
Control	0	0	26,750 ± 1658	-	-	
Antimicrobial gel	25	30	375 ± 35	1.9	98.60	<0.001
	25	120	225 ± 177	2.1	99.16	<0.001
	50	30	0 ± 0	4.4	99.99	<0.001
	50	120	0 ± 0	4.4	99.99	<0.001

**Table 2:** Ability of vapours of essential oils to reduce the infectivity of aerosolised murine hepatitis virus.

Sample	Amount of Gel (g)	Evaporation Time (min)	Number of Plaque Forming Units (PFU/mL)	Log_10_ Reduction	% Reduction	*p*-Value
Control	0	0	1152 ± 354	-	-	
Antimicrobial gel (no neutraliser)	10	10	596 ± 149	0.3	48.26	0.002
Control	0	0	1396 ± 240	-	-	
Antimicrobial gel (no neutraliser)	10	20	650 ± 101	0.3	53.44	0.001
Antimicrobial gel (with neutraliser)	10	20	930 ± 142	0.2	33.48 *	0.001

*, no difference in activity when no neutraliser was added (*p* = 0.078).

**Table 3:** The ability of vapours of essential oils to reduce the numbers of aerosolised *E. coli* K12.

Sample	Amount of Gel (g)	Evaporation Time (min)	Number of Bacterial Colony Forming Units (CFU/mL)	Log_10_ Reduction	% Reduction	*p*-Value
Control (no neutraliser)	0	0	86 ± 14	-	-	-
Antimicrobial gel (no neutraliser)	10	10	42 ± 20	0.3	51.16	0.001
Control (with neutraliser)	0	0	139 ± 46	-	-	
Antimicrobial gel (with neutraliser)	10	10	99 ± 47	0.1	28.77 *	0.018

*, significantly less activity (*p* = 0.01) compared to no neutraliser.

**Table 4:** The ability of the antimicrobial gel vaporised for 10 min to reduce the numbers of aerosolised *A. flavus* spores.

Sample	Amount of Gel (g)	Evaporation Time (min)	Number of Fungal Spores as Colony Forming Units (CFU/mL)	Log_10_ Reduction	% Reduction	*p*-Value
Control (no neutralizer)	0	0	231 ± 42			
Antimicrobial gel (no neutralizer)	10	10	65 ± 7	0.6	71.86	0.008
Control (with neutralizer)	0	0	170 ± 77	-	-	
Antimicrobial gel (with neutralizer)	10	10	57 ± 23	0.5	66.47 *	0.001

*, no difference in reduction of fungal cells compared to no neutraliser (*p* > 0.08).

## Data Availability

Data available upon request.
